# [Expression of Concern] Long non-coding RNA OIP5-AS1 promotes the progression of esophageal cancer by regulating miR-30a/VOPP1 expression

**DOI:** 10.3892/ol.2026.15752

**Published:** 2026-07-08

**Authors:** Jiajun Xu, Zhixi Chen, Zheng Fang, Shixiong Chen, Ying Guo, Xianfeng Liu, Kai Chen, Shengjia Chen

Oncol Lett 22: 651, 2021; DOI: 10.3892/ol.2021.12912

Following the publication of the above paper, it has been drawn to the Editor's attention by a concerned reader that a pair of data panels in Fig. 2D and F showing the results of migration/invasion assay experiments contained an overlapping section, such that data panels which were intended to show the results of differently performed experiments had been derived from the same original source. In addition, three of the data panels in Fig. 7C and E, also showing the results of cell migration/invasion assay experiments, appeared subsequently in a paper submitted to the journal *Journal of Cancer* that featured the author Zhixi Chen in common. Furthermore, upon performing an independent analysis of the data in this paper in the Editorial Office, it came to light that western blot data featured in Fig. 6E appeared subsequently in a paper featuring no authors in common in the journal *Frontiers in Pharmacology*, and also in another paper featuring the author Zhixi Chen in common that had already been accepted for publicaton in the journal *Oncology Letters*.

The authors have been contacted by the Editorial Office to offer an explanation for these apparent anomalies in the presentation of the data in this paper, and we are awaiting their response. Owing to the fact that the Editorial Office has been made aware of potential issues surrounding the scientific integrity of this paper, we are issuing an Expression of Concern to notify readers of this potential problem while the Editorial Office continues to investigate this matter further.

